# Physiological Psychology

**Published:** 1858-04-01

**Authors:** Robert Dunn


### Art. V.—PHYSIOLOGICAL PSYCHOLOGY.

### No. V.

BY ROBERT DUNN, F.R.C.S. ENG.

(*Continued from No. VI., p.* 367.)

*Recapitulation.*—Consciousness we have viewed in the light of an *ultimate fact,* beyond which we cannot go,—as the *distinguishing* attribute of animal life; and self-consciousness as the *primary condition of intelligence,*—in a word, as *mental existence.* It is, in fact, equivalent to the knowledge which we possess of our own personal identity, for it is implied in every sensation which we experience, and in every mental act that we perform—in *feeling, perceiving, thinking,* and *willing. Consciousness* and *immediate knowledge* are universally convertible, and psychology itself is only developed consciousness. Reid and his follower, Dugald Stewart, were clearly in error in restricting the function of consciousness to that of a *particular faculty*, co-ordinate with the other intellectual powers, instead of regarding it as *the universal condition of intelligence*. For—

"In consciousness, as the original spontaneity of reason (*νοῦς*, *locus principiorum*), are revealed the premordial facts of our intelligent nature. Consciousness is the fountain of all comprehensibility and illustration, but *as such* cannot itself be illustrated or comprehended. To ask how any fact of consciousness is possible, is to ask how consciousness itself is possible; and to ask how consciousness is possible, is to ask how an intelligent being like man is possible."*

Now, we can best conceive of consciousness, as "one and indivisible" in relation to time, as an incalculably rapid succession of acts or states, and as passing from the moment of birth—the helplessness of infancy—to the maturity of age, through a progressive series of developments—through the different phases of sensational, perceptive, and intellectual consciousness. TO FEEL, TO PERCEIVE, AND TO THINK, or, in other words, *sensation, perception*, and *intellection*, are distinctly progressive stages in our psychological progress. They are the three great and distinguishing phases of mental activity and development, under which are comprised all our psychological phenomena, of whatever kind; and they each severally have and require, for the manifestation of their respective phenomena in this life, *a distinct organic instrumentality*, of corresponding elaboration and complexity, in the nervous apparatus of the cerebro-spinal system of man.

*Sensori-motor, consensual and instinctive feelings and actions*, are the phenomena which formulate the sensational consciousness; to these are superadded, *ideation and volition, with their associates, memory and emotional sensibility*, as the essential phenomena of the perceptive consciousness; and to these, *imagination, imitation, articulate speech or language, ratiocination, and the processes of thought and reflection*, as the distinguishing attributes of the intellectual consciousness.

But to ask a *reason*, as Sir William Hamilton has justly remarked, for the possibility of our *intuitions*, sensational or perceptive, above the *fact of their reality or consciousness*, "betrays," as Aristotle has truly said, "*an imbecility of the reasoning principle itself;*" for the facts, *as ultimate*, are inexplicable. "What we do know of self, or person, we know only as given in the consciousness: there is revealed to us, as an ultimate fact, a *self* and a *non-self;* each given as independent—each known only in antithesis to the other. And no belief can be more *intuitive, universal, im-*

* Sir William Hamilton.—*Vide* "Edinburgh Review," vol. lii. p. 176.

*mediate*, or *irresistible*, than that this antithesis is real, and known to be real; and no belief is therefore more true."*

Whatever may be the notion entertained of the abstract nature or essence of mind, one thing is manifestly obvious, that the sensori-motor phenomena—in other words, that *sensibility* and *motility*—indicate its primordial points of contact with the external world or nature. And as to the universality of *instinctive endowments*, Sir Henry Holland has well observed:—

"Wherever there is organization, even under the simplest form, there we are sure to find *instinctive action*, more or less in amount, destined to give the appropriate effect to it. This is true throughout every part of the animal series, from men and the quadrumana down to the lowest form of infusorial life. When we consider how vast this scale is—crowded with more than a hundred thousand recognised species, exclusively of those which fossil geology has disclosed to us—we may be well amazed by the profuse variety of *instinctive action;* as multiplied in kind as are the organic forms with which it is associated, and all derived from one common POWER—that of *instinct*."†

But amazing as this may be, are we not still more in "wondering mazes lost," when we reflect upon *all* the endowments of the *primordial cell* (vegetable or animal), *with its granular nuclei*, microscopically minute, and upon the *community* of *function* of its ultimate constituents; bearing in mind, at the same time, that within this *solitary cell* are potentially contained not only the *instinctive activities* above indicated, ranging from the infusoria up to man, but the perceptive, the affective, and the intellectual faculties of every class,—the god-like attributes of man himself, and even distinguishing peculiarities and idiosyncracies hereditarily transmitted from parent to offspring?

Of the three forms of mental activity—the sensational, perceptive, and intellectual, and under which all our varied psychological phenomena arrange and group themselves—*self-consciousness* is the earliest, and consequently the lowest, phase of development; for in it the mind at first exists in a state of bare receptivity. The senses, indeed, come into play from the moment of birth, and soon acquire the utmost perfection of which they are capable; but the *intelligence* is purely sensational, the *feelings* are simply those of pleasure and pain, and the *impulses to action* are innate and instinctive. In perceptive or *world-consciousness*, an increasing amount of mental activity obtains, arising from the conflict of the perceptive faculties of the mind with the external world or nature. Indeed, all our *immediate knowledge*, of whatever kind, is intuitive, and has its origin in perceptive experience.

* *Vide* "Edinburgh Review," *ante cit.*

† Sir Henry Holland's "Chapters on Mental Physiology."

Here we have superadded to sensational, the *perceptive intuitions, ideation, and the cognition of outward realities;* to sensorial feelings, the *emotional and moral sensibilities;* and to innate and instinctive impulses, *volitional powers* and *intelligent actions.* But it is in the *intellectual consciousness* that the mental activity reaches its culminating point, in the region of *representative knowledge.* It is here, through imitation, imagination, and ratiocination, that the mind attains to its highest phase of development—grasping, through the range of the intellectual and reflecting faculties, *abstract ideas,* and *necessary and universal truths,* and finding articulate utterance and expression for them through the faculty of speech in language.

Now, nervous actions are of a threefold character—*physical, sensory,* and *voluntary.* There are—

1st. The physical and reflex actions of the excito-motory system, which occur *without sensation;*

2ndly. The sensori-motor — the consensual and instinctive actions of the sensational consciousness; and

3rdly. The voluntary—the volitional, emotional, and intelligent actions of the perceptive and intellectual consciousness.

We recognise and detect the first of these, the physical and reflex, exclusively *only* in the lowest animal organisms. They are essentially automatic, and occur *without sensation.* The sensori-motory actions are typical of animal life, and in conjunction with the first, or excito-motory, characterise the whole of the invertebrate sub-kingdom; whilst in the vertebrate series, to the first and second, the third, or purely voluntary, are superadded, and constitute the distinguishing feature of the cerebro-spinal system.

Accordingly, the nervous system of man, from the nature of its office, and its functional endowments, admits of a threefold division also, into—

1. The physical or excito-motory, and reflex—*the true spinal system of Dr. Marshall Hall;*

2. The nutritive and secretory, or ganglionic system; and

3. The sentient, psychical, and voluntary, or cerebro-spinal system.

But it is only with the last of these, the cerebro-spinal system, that we are now more immediately concerned; for the physical or excito-motory phenomena are *without* the domain, and *beyond* the control of intelligence,—and the reciprocal relations of the ganglionic and the cerebro-spinal systems have but an indirect though most important practical bearing upon our present subject of inquiry. As we have said, in its totality, the nervous apparatus of the cerebro-spinal system comprises the *organic instrumentalities* through which the phenomena of sensation, perception, and intellection are manifested in this life; in other words, the nervous centres of the sensational, perceptive, and intellectual consciousness. Now, we have seen that the sensory ganglia are the seat of the sensational consciousness of whatever kind, and not only of sensorial, but of emotional feeling; and that the cranio-spinal axis, and the corpora striata at its summit, are the centres and source of all the movements of the body—reflex, sensational, emotional, and volitional; so that the nervous apparatus of the sensational consciousness—the system of automatic life and instinctive action, subservient to sensation, and to those consensual and instinctive actions which are indissolubly linked on with sensations—consists of the spinal axis and nerves, the medulla oblongata, and the chain of sensory ganglia, including those of the special senses at its summit. For, as I have before observed, if we follow up the cranial prolongation of the spinal cord, the medulla oblongata, into the fibrous strands of which we see imbedded *the respiratory, auditory*, and *gustatory ganglia*, and carefully trace out its ramifying branches, we find it sending off distinct fasciculi of fibres to the ganglionic centres at its summit, to the cerebellum, the corpora quadrigemina, the thalami optici, the corpora striata, and to the peduncles of the olfactory ganglia, and thus to *the sole exclusion of the cerebrum*, which is an organ superimposed and superadded, and whose connexions are strictly *commissural*, the whole series of the ganglia of the cerebro-spinal system, including those of the spinal senses, are in direct fibrous connexion with the cranio-spinal axis, forming with it, as an aggregate or whole, *the sensorium commune, or great circle of sensational consciousness, and of consensual and instinctive action.*

Now, as the functions of an *independent centre of action*, seated in a *distinct nervous apparatus*, the phenomena of the sensational consciousness are not to be confounded with volitional or intelligent actions. But upon this nervous apparatus of the sensational consciousness, for the purpose of combining and associating, in the development of the active powers of his mind, instinctive impulses, sensational feelings, emotional and moral sensibilities, with the higher intellectual activities, and for offices and purposes the noblest and most exalted in the economy of man, there is superimposed and superadded—the cerebrum, or great hemispherical ganglia, and which, in its totality, is the seat of the instruments or organs both of the perceptive and of the intellectual consciousness. For if there be one point in the physiology of the brain more unequivocally demonstrated than another, it is this—that these ganglia are the instruments of intellectual action and volitional power; and that, wherever they exist, even in their simplest rudimentary condition, when compared with their complex and full development in man, there we invariably find manifested the essential phenomena of the perceptive consciousness—*ideation, memory*, and *volition.*

Restricting the functions of the cerebrum solely to perceptive and intellectual operations, *to the entire exclusion of sensation,* Dr. Carpenter, to my mind, has fully established the composite nature of the animal propensities and social affections, and of the emotional, moral, and religious feelings of man; and has admirably shown, that in the exercise of each there is a *perceptive or ideational element,* as well as *sensorial feeling,* involved. And here it is worthy of remark, that that sagacious metaphysician, Mr. James Mill, in his contemplations on human mind, apart from all physiological considerations, had previously arrived at the same conclusions. The separation and localization, within the encephalon, of the nervous centres of sensation and of ideation, of feeling and of thought, is a real and an acknowledged step in advance on the physiological psychology of man, and it is one which has yet to be fully appreciated, in all its variety of bearings, in relation to the practice of psychological medicine.

Now, although the cerebrum, in its totality, is indisputably the seat of the organs both of the perceptive and of the intellectual consciousness, perception and intellection are not to be confounded with each other.

*To perceive and to think are distinct mental processes,* and they have—for they must require—distinct organic instrumentalities for the manifestation of their respective phenomena. Perception is but one and the first step above sensation; its intuitions are closely interwoven with feeling, and are often, indeed, intensely felt. It is intermediate between sensation and intellection, and it is the portal to intellectual action, for the intuitions of its faculties furnish the *pabula of thought.* But intellection is the highest, the crowning phase of mental development and introspective or reflective consciousness—the distinguishing attribute of humanity—and as to *feeling,* it finds no place in the constitution of abstract ideas, or in the processes of logical reasoning.

Perception speaks to us from *without,* and intellection from *within;* for whilst, in the perceptive consciousness, ideation is affected, in response to impressions made upon us from *without,* by virtue of the primeval harmony which exists between the perceptive faculties of the mind and external nature,—in intellection, the *mental process is different and reversed,* and the mind, separating itself from outward restraints, and impelled by the inherent activity of its intellectual faculties and reflecting powers, embodies idealized impressions and perceptive intuitions—its inward images and representative ideas, in *objective realities.* Thus symbolized or objectified, they are removed from the region of immediate and perceptive experience, and exist in the mind as independent intellectual realities, and become *fixed* and *definite objects of thought*, and which can be placed at pleasure either within or without the consciousness of the moment. In this way it is that the mind, impelled by the *imitative faculty*, by means of the hands and chisel, moulds, forms, and fashions images of the objects of nature, into which it has embodied its own generalized ideas; and so, again, urged on and impelled forward by the same imitative faculty, by means of the hands and the pencil, it delineates and produces pictorial representations of the idealized objects;—*such were the hieroglyphics of old.* But still the mind cannot be said to have achieved its first step *in the freedom of human thought* until it has created, invented, and constructed its own sign, phonetic or visible, for the embodiment of the intellectual idea; and such, as we have seen, is *language*—for, in reality, "*it is idea objectified.*"

It is important, physiologically as well as psychologically, to bear in mind the *distinction* between *immediate* and *representative* knowledge, and the difference in *their origin or source.* For all our immediate knowledge, of whatever kind, is *intuitive,* and has its origin in perceptive experience—in the direct intuitions of the perceptive faculties; and all representative knowledge is the *product* or *creation* of the mind's intellectual and reflecting powers.

Now, in the structure of the cerebrum there are manifestly two well-marked and distinct series of convolutions. *There is a longitudinal, and there is a transverse series;* and my own mind rests on the conviction, that the *functions* of these two distinct series of convolutions are *different;* and that the former, or longitudinal series, constitutes the nervous apparatus of the perceptive, and the transverse series that of the intellectual consciousness.

But, besides these two distinct series of convolutions, there is a third or commissural series in the cerebrum, and through the instrumentality of which the *intuitions* of the perceptive are brought into association with the *higher activities* of the intellectual consciousness. Such are *the internal anastomosing convolutions*, the third order of Foville, which connect the *ourlet*, or great internal, with the transverse convolutions on the surface of the cerebrum—the common central organs of the perceptive, with those of the intellectual consciousness.

Foville, as we have seen, has invested the *locus perforatus*, or quadrilateral spot, with peculiar interest; considering it, as he does, the nucleus, or starting-point, from whence all the other convolutions of the hemisphere are evolved. Nor can the fact be denied, that the *ourlet* of Foville, or great internal—the primitive basement convolution of the hemisphere—may literally be said to spring out of, or to be evolved from, the *locus perforatus*, and that all the other longitudinal convolutions of the hemisphere are directly connected and associated with this primitive basement convolution; forming as a whole, in the aggregate, in my opinion, the nervous apparatus of the perceptive consciousness. But it is equally true that the transverse series of convolutions on the surface of the cerebrum have no direct connexion either with the *locus perforatus*, or with the *ourlet* of Foville. They are a distinct series of convolutions; and it is through a system of internal anastomosing convolutions that these transverse convolutions are brought into association with the central organs of the perceptive consciousness. They constitute, in my mind, the nervous apparatus of the intellectual consciousness, for they are essentially human; and it is only where they do exist, and in the ratio or proportion of their existence, among the lower animals, that we find and detect any traces of ratiocination and of intellectual action.

Foville has traced out and demonstrated the connexions of all the other primitive longitudinal convolutions of the cerebrum with the basement convolutions of the hand; and Plate XII. of the Quarto Atlas to his "Traité Complet de l'Anatomie, de la Physiologie, et de la Pathologie du System Nerveux-Cerebro-Spinal," is "destinée montrer les rayonnements de l'ourlet fibreux dans le circonvolutions de la face interne de l'hemisphere." In this plate, with the great commissure divided in the mesial line, there is seen, above the corpus callosum, a vertical section of the great internal convolution—the *ourlet* or hem of the hemisphere—its concentric circumference—surrounding it internally like a riband, and attached at each extremity to the *locus perforatus*. The great marginal convolution of the longitudinal fissure is seen forming the eccentric or outer boundary of the hemisphere; and between these, crossing the internal surface of the hemisphere, are displayed the convoluted branches which unite them with the anterior, middle, and posterior longitudinal convolutions of the brain, establishing the connexions, and forming a sort of anastomosis of the basement convolutions with all the other primitive convolutions, and with the *transverse convolutions*, on *the convex surface of the cerebrum*. In Plate X. is represented the external surface of the hemisphere. All the convolutions of the convexity of the hemisphere are seen running, from the convolution around the fissura Silvii to that which encircles the hemisphere, the great marginal one. The transverse superciliary, medio-parietal, and occipital convolutions are displayed, and, besides their connexions with the two convolutions of the second order, their anastomosis with each other.

The great internal convolutions, as I have said, are clearly the primitive basement convolutions of the hemispheres; and we recognise their homologues in the thin laminæ of vesicular matter which encrust the corpora striata in the brain of the fish. Forming, as they do, the concentric or inner circumference of the hemispheres, as the great marginal convolutions do their outer boundary, must they not necessarily be the primary and common portals to intellectual action—the great central organs of the perceptive consciousness—the seat of ideation, memory, and volition? For, be it remembered, that it is in the case of the fish, where their representatives are reduced to mere laminæ or crusts, covering the corpora striata, that we have the earliest instance, and the first clear and distinct evidence, of the exercise of *perception*, *memory*, and *volition*, as opposed to mere consensual and instinctive actions. These convolutions of the band constitute the distinctive and boundary lines of demarcation between the *sensory* and *perceptive ganglia* of the encephalon—between sensation and ideation. They are, in fact, the common portals to intellectual action and volitional power—the seat of *ideation*, *memory*, and *volition*. It is *here* that sensory impressions—the intuitions of all the organs of the special senses—are *idealized* and *registered*, *perceived* and *associated*, and that the *ideation* or *world-consciousness* of external existences—the things which we see, feel, taste, and smell—is effected; and it is from *here* that the mandates of the will issue. We have seen that with these central organs and fundamental convolutions are directly connected and associated all the other longitudinal convolutions in the anterior, middle, and posterior lobes of the brain, administering to the several perceptive ideational activities of man, and to the development of his composite nature as an animal and social, a moral and religious, as well as an intellectual being.

Now may it be fairly concluded, that the intuitions not only of all the special senses, but also of all the perceptive faculties, are *perceived* by us through the central organs of the perceptive consciousness? Most certain it is that we have an æsthetic sense of the true, the beautiful, and the good,—moral intuitions of right and wrong, and emotional of awe, veneration, and reverence, which come to us before all teaching,—and indeed, that the elements of all our immediate knowledge, physical, moral, and religious, have their origin or source in perceptive experience.

To determine the special functions of the primitive convolutions is the great problem of physiological psychology; and although something may be said to have been done towards its solution, much remains to be accomplished, for the problem is virtually unsolved. Nor can this be a matter of surprise, when we consider its conditions and requirements.

The natural history of the development of the cerebrum, throughout the whole vertebrate sub-kingdom, is but the first step in the process, and it is one that has been or may be accomplished; but the far more difficult task remains,—a work of labour as necessary as it is difficult,—that of studying the characters, habits, and behaviour of the animals throughout the series,—their animal and social propensities, and intellectual activities, in connexion with their respective cerebral developments; and how few are there among us who possess the mental endowments and requisite qualifications, and can command opportunities, even on a limited scale, for such an undertaking. But, "*Nil, sine magno labore, debit mortalibus;*" and with such active labourers in the field as Holm, Vimont, and Frederick Cuvier have proved themselves to be, great things will assuredly, in the process of time, be accomplished in furtherance in this direction of the study of the physiological psychology of man.*

* Professor Owen has proposed a fourfold primary division of the mammalia, based upon four leading modifications of the structure of the cerebrum, under the following designations:—

1. *Lyencephala* (λύω, to *loose;* εγκεφαλος, the brain), the loose-brained implacentals, in which the great transverse commissure, or corpus callosum, is wanting—such are the marsupialia and monotremota.

2. *Lessencephala* (λισσὸς, *smooth*), the smooth-brained placentals, where the corpus callosum is present, but the brain is not convoluted—such are the rodentia, insectivora, &c.

3. *Gyrencephala* (γυρόω, to wind *about*), in which the superficies of the brain is folded into more or less numerous gyri, or convolutions, of which among the higher are the quadrumina and carnivora. The mammalian modification of the vertebrate type attains its highest physical perfections in the gyrencephala, as manifested by the bulk of some, by the destructive mastery of others, by the address and agility of a third order. And through the superior psychological faculties—an adaptive intelligence predominating over blind instinct—which are associated with the higher development of the brain, the gyrencephala afford those species which have ever formed the most cherished companions and servitors, and the most valuable sources of wealth and power to mankind.

4. *Archencephala, Homo* ('Αρχω, to *overrule*). "In man," says Professor Owen, "the brain presents an ascensive step in development, higher and more strongly marked than that by which the other sub-classes are distinguished. Not only do the cerebral hemispheres overlap the olfactory lobes and the cerebellum, but they extend in advance of the one, and farther back than the other. Their posterior development is so marked that anatomists have assigned to that part the character of a *third* lobe; *it is peculiar to the genus Homo,* and *equally peculiar is the 'posterior horn of the lateral ventricle,' and the 'hippocamus minor,' which characterize the hind lobe of each hemisphere.* The superficial grey matter of the cerebrum, through the number and depth of the convolutions, attains its maximum of extent in man.

"Peculiar mental powers are associated with this highest form of brain, and their consequences wonderfully illustrate the value of the cerebral character, according to my estimate of which I am led to regard the genus *Homo* as not merely a representative of a distinct order, but of a distinct sub-class of the mammalia, for which I propose the name of Archencephala."†

† *Vide* a paper by Professor Owen, 'On the Characters, Principles of Division, and Primary Groups of the Class Mammalia," read before the Linnæan Society of London, Feb. 17th, and April 21st, 1857, and in the "Journal of the Proceedings of the Linnæan Society, for June, 1857," vol. ii. No. 5.

The facts, indeed, of developmental anatomy, comparative and human, point to the most important deductions, for they indisputably prove—firstly, that the perceptive faculties of our physical experience or knowledge, subservient to our cognition of external objects, their sensible qualities and physical attributes, the phenomena of their action, or events, and their relative relations, arrangement and number, &c., must have their "local habitation and abode" in the convolutions of the anterior lobes; secondly, that the posterior lobes, as exclusively human, must necessarily be the seat of the exclusively human affections, and administer to our social affections; and thirdly, the inference appears to be legitimate, that the convolutions of the middle lobes are the seat of the personal affections of the ego, and of the social, moral, and religious intuitions—the distinguishing attributes of man.

The general harmonious accordance of these deductions with the multiplied cranioscophical observations of Gall, Spurzheim, and Combe, speaks in favour of their foundation in truth and nature; and I think it may be legitimately inferred that in the primitive basilar convolutions are seated the organs of the faculties subservient to the *formation of the inferior region* of the true or conscious mind. Thus, on the lowest plane of cerebral development, and of which we may recognise the analogues in the inferior vertebrata, the perceptive apparatus seems limited to the basement or internal convolutions, with their anterior and basilar connexions; that is, to the common central organs of the perceptive consciousness,—the seat of ideation, memory, and volition,—to the anterior perceptive organs, through the instrumentality of which, by the inlets of the special senses, we acquire a knowledge of the sensible qualities and physical attributes of external existences,—and to those basilar organs which administer to the preservation and maintenance of animal life. The *love of life* is paramount; and around the organs of the alimentative propensity are marshalled and associated those of the propensities subservient to the defence, protection, and conservation of existence. It may, indeed, be fairly inferred that the intuitions of the special senses and their allied feelings, appetites, and instincts, form the chief and predominant part of the mental life of the inferior vertebrata, while at the same time it must not be forgotten that these, too, constitute the inferior region of the true or conscious mind, and enter largely into the complicated web of human existence. Again, on a higher plane of development, and of which, too, we may recognise the analogues among the highest mammalian and quadrumanous groups, the longitudinal convolutions are carried upwards above the lower perceptive organs, and prolonged backwards even beyond the median lobes, and the perceptive apparatus is thus proportionately elaborated and extended.

Contrasting the endowments of the higher mammalia with the ruminants, in accordance with this is the remark of Leuret when describing the convolutions of the Indian elephant. "Suppose," says he, "that all the superior convolutions, and the prolongation of the great internal convolution which is united to them, to be obliterated, then the fourth anterior convolution might be united to the fourth posterior,—the third to the third,—and we should have one of the groups of convolutions of the brain of the ruminants and solipedes." It is through these superior perceptive organs that we rise above the bare perception of external objects, their sensible qualities and physical attributes, to that of the differences and relations of things, their order or arrangement, and number, and to the phenomena of their action, *or events*, with the adjuncts of time and place. The higher individual or personal affections, too, such as the *love of self*, or *self-esteem;* the *love of approbation;* and *love for others*, or *benevolence*, are brought into play. But there is a still higher plane of perceptive development *exclusively human*, in which the towering longitudinal convolutions reach the fulness of their evolution backwards, and the nervous apparatus of the perceptive consciousness its most elaborate and complete development. The moral and religious intentions are the sole *prerogatives of man*, and they constitute an immutable distinction between him and the whole animal creation. In man's moral and religious attributes the lower animals do not participate. Equally destitute are they of those enduring, tender, and endearing relations which are the charm of his existence here.

And now, may it not be fairly concluded, from the close proximity and intimate association of this highest plane of perceptive development with the *transverse series of convolutions*, that the exalted, pure, and holy intuitions of the one will be directed, guided, and strengthened by the dominating influence of the noblest faculties of the other; that through the latter, our æsthetic sense of the true, the beautiful, and the good will not always end in fruitless aspirations, but fructify; and that, also, through them, while they control, direct, and strengthen our moral intuitions of right and wrong, and our emotional of awe, veneration, and reverence, we will be led to see clearly the *basis* upon which *moral obligation* rests, and religion will become to us "a reasonable service," and ours an intelligent, voluntary, and cheerful dependence upon an *all-perfect Being*, infinite in wisdom, power, and goodness? It is in this association of the pure and elevating moral and religious intuitions with intellectual power that the *true greatness* of the human character consists; and, in fine, that from the joint operation of the highest perceptive faculties with the reasoning powers, from *observation* and *experiment,* result the creation of science and the achievements of science.

"For the proper function of reason is to create knowledge or science. The *understanding* alone can never do this. It can analyse, distinguish, form concepts, construct propositions, weave them into arguments—perform, in a word, any formal process within the data furnished to it—but it can never go beyond the barriers of its own definitions. When, however, we grasp a truth by the power of reason, on the other hand it implies far more than the attainment of a bare definition of it. It implies that we have penetrated to the centre; that we can trace its pedigree in the world both of matter and form; that we can regard it as one link in a connected chain, of which we are able to *tell* the antecedents and *foretell* the consequents; that we can recognise it, in fine, as a particular manifestation of some great and universal law, the operation of which we have learned to comprehend and apply."*

In connexion with the *processes of thought*, we have evidence of an *automatic action* of the intellectual faculties, *unattended with consciousness,* designated *unconscious cerebration* by Dr. Carpenter, who was the first to bring it under the notice of psychological inquirers, and who, in support of its existence, has obtained the confirmatory experience of two of the most distinguished metaphysicians and profound logicians of the age—the late Sir William Hamilton and Mr. John Mill.

"Most persons," says Dr. Carpenter, "who attend to their own mental operations are aware that, when they have been occupied for some time about a particular subject, and have then transferred their attention to some other, the first, when they return to the consideration of it, may be found to present an aspect very different from that which it possessed before it was put aside, notwithstanding that the mind has since been so completely engrossed with the second subject as not to have been consciously directed towards the first in the interval. Now, a part of this change may depend upon the altered condition of the mind itself, such as we experience when we take up a subject in the memory, with all the vigour which we derive from the refreshment of sleep, and find no difficulty in overcoming difficulties and disentangling perplexities which checked our farther progress the night before, when we were too weary to give more than a languid attention to the points to be made out, and could use no exertion in the search for these solutions. But this by no means accounts for the *entirely new development* which the subject is frequently found to have undergone when we return to it after a considerable interval—a *development* which cannot be reasonably explained in any other mode than by attributing it to the intermediate activity of the cerebrum, which has, in this instance, *automatically evoked the result without our consciousness.*†

* Morrell's "Psychology."
† Dr. Carpenter's "Human Physiology," 5th edition.

To the same effect is the following remark of Sir Benjamin Brodie:—

"It has often happened to me to have been occupied by a particular subject of inquiry—to have accumulated a store of facts connected with it, but to have been able to proceed no further. Then, after an interval of time, without any addition to my stock of knowledge, I have found the obscurity and confusion in which the subject was originally enveloped to have cleared away, the facts seemed all to have settled themselves in their right places, and their mutual relations to have become apparent, although I have *not been sensible* of having made any distinct effort for that purpose."*

And such, from personal experience, I conceive to be the common experience of every thinking mind. I have already observed† that *automatic* or *reflex action* is not peculiar to the true spinal system; but, on the contrary, that it is the common attribute of the sensori-motor, emotional, and cerebral systems, and that Dr. Laycock was the first to connect it with the cerebrum. Dr. Carpenter goes on to remark:—

"Strange as this phenomenon may at first sight appear, it is found, when carefully considered, to be in complete harmony with all that has been affirmed respecting the relation of the *cerebrum* to the *sensorium*, and the independent action of the former. Looking at all those automatic operations by which results are evolved without any intentional direction of the mind to them, in the light of *reflex actions* of the cerebrum, there is no more difficulty in comprehending that such reflex actions may proceed without our knowledge, so as to evolve *intellectual products*, when those results are transmitted to the sensorium, and are thus impressed on our consciousness, than there is in understanding that impressions may excite muscular movements through the *reflex* power of the spinal cord, without the necessary intervention of sensation. In both cases, the condition of this mode of independent operation is, that the *receptivity* of the sensorium shall be suspended *quoad* the changes in question, either by its own functional inactivity, or through the temporary engrossment by other processes."

Dr. Carpenter extends the same *unconscious* or *automatic reflex action* to the feelings of the *emotional* states, and says—

"That our feelings towards persons and objects may undergo most important changes without our being in the least degree aware, until we have our attention directed to our own mental state, of the alteration which has taken place in them. A very common but very characteristic example of this kind of action is afforded by the powerful attachment which often grows up between individuals of opposite sexes, without either being aware of the fact; the full strength of this attachment being only revealed to the consciousness of each when

* "Psychological Inquiries," by Sir B. C. Brodie.
† *Vide* page 402, vol. ix. of this Journal.

circumstances threaten a separation, and when each becomes cognizant of the feelings entertained by the other. . . . .

"We continually speak of the 'feelings' which we *unconsciously* entertain towards another, and of our not becoming aware of them until some circumstances call them into activity; so that it would seem as if the material organ of these feelings tends *to form itself* in accordance with the impressions which are habitually made upon it, and that we are as completely unaware of the changes which may have taken place in it, as we are of those by which passing events are registered in our minds (in the memory), until some circumstance calls forth the conscious manifestation, which is the 'reflex' of the new condition which the organ has acquired." *

To the category of the *ideo-motor phenomena* belong, as Dr. Carpenter has shown, a variety of aberrant actions, bordering on insanity, of which the history of mankind in all ages furnishes us with abundant examples; and among the most recent, but not the least remarkable instances, is the *table-turning* and *table-talking* epidemic which spread through almost the whole civilized world in 1852-3; and to his able disquisition on the subject the reader is referred.†

After the mind has been pondering over the perplexities of a difficult subject of thought, that the *automatic* or *unconscious reflex action* of the intellectual and reflecting organs should, during a period of repose—that of profound sleep—*evolve clear ideas and new developments of thought* in connexion with the subject, may be truly wonderful; but is it more wonderful that during sleep, when the controlling influence of volition is suspended, a mathematical problem should be solved, than that a poetical fragment like the "Kublakhan" of Coleridge should be composed? Condorcet saw, in his dreams, the final stage of a difficult calculation which had puzzled him during the day; and Condillac, when engaged in his "Cours d'Etude," frequently developed and finished a subject in his dreams, which he had broken off before retiring to rest. Coleridge says of himself, that his fragment, "Kublakhan," was composed during sleep, "the images rising up before him as things, with a parallel production of the corresponding expressions, without any sensation or consciousness of effort." The imagination, it is true, is prone to run riot when the controlling influence of the will is withdrawn, and, as in dreams,—

"To combine a medley of disjointed things—
A court of cobblers, and a mob of kings."

At all times, indeed, the *imaginative* are less amenable to the dominion of volition than are the *reasoning processes;* but still

* Dr. Carpenter's "Human Physiology," pp. 609-10, 5th edition.
† Ibid. pp. 610-18, 5th edition.

we must bear in mind the mental relations of the imaginative faculty and the reasoning powers.

Out of the fanciful combinations and groupings of external objects, *new conceptions* are formed; and by the imaginative faculty, ideality, we are placed in scenes, circumstances, and relations in which our actual experience has never placed us, and from which, in consequence, as *new sources* of thought, *new conceptions arise.* But while these new creations may bear strongly the impress of the æsthetic and emotional character and tendencies of our minds, the highest efforts of the creative faculty involve equally the agency of the intellectual powers—of collocation, analysis, and comparison—to achieve their loftiest triumphs. And thus, while, on the one hand, *ideality* is dependent upon the intellectual powers for the development of its highest and sublimest flights; so, on the other, is the *understanding* indebted to the imaginative faculty for those *ideal combinations and conceptions* which, independently of their artistic value and importance, are seen to be so operative in the common affairs of human life,—"suggesting those pictures of the future which are ever before our eyes, and are our animating springs of action, with those visions of enjoyment, never perhaps to be realized, and their prospects of anticipated evil, that often prove to be an exaggeration of the reality—prompting the investigations of science, that are gradually unfolding the sublime plan on which the UNIVERSE is governed, and leading to a continual aspiration after those higher forms of moral and intellectual beauty which are inseparably connected with purity and love."

*Conclusion.*—In the attempt, however crude and cursory, which in these papers has been made towards an exposition of some of the leading points of Physiological Psychology, while I may put forward some claim to originality of view, in respect to the nervous apparatus of the perceptive and intellectual consciousness, I am free to confess that I have not hesitated to adopt the opinions and sometimes the language of others, but never intentionally without due acknowledgment, and at all times in the hope of exciting the attention of others, and of rousing into activity the energy of other minds of higher endowments, possessing better opportunities and more leisure for the prosecution of such an interesting and important subject of inquiry.

The establishment of the "Psychological Journal" has given a new impetus to such investigations; and the philosophy of the mind, like the philosophy of nature, is now cultivated in a manner worthy of its objects. The *phantasms* of Aristotle, the *animal spirits* of Descartes, and the *vibrations* of Hartley, alike have passed away as physiology and psychology have progressed.

Still, we are under great obligations to Hartley;* for however unfruitful and visionary his "Doctrine of Vibrations" may be, he appears to have been the *first* in this country, and Bonnet on the Continent, who brought and employed a sound and experimental knowledge of the human constitution to the attempt to discover the physical conditions of sensation and intelligence. I am fully aware how important it is to keep in view the distinctive boundaries of physiology and psychology, and that it is only in their correlations—when certain *phenomena of observation* are found uniformly to co-exist with *certain phenomena of consciousness*—that their direct bearing upon each other can be really established. I am, nevertheless, impressed with the idea that *physiological* bears to *medical psychology* a relationship analogous to that which physiology does to pathology, so that a clear comprehension of the principles of the former appears to me to be essentially necessary for a proper and full appreciation of the abnormal and morbid phenomena of the latter. It has been well observed by Feuchtersleben, in his admirable treatise,—

"Where psychical phenomena appear *abnormal*, there is *mental disorder*, which has its root in the mind, so far as this is manifested through the sensual organs; and has its root in the body, so far as this is the organ of the mind. To search after phenomena in which these relations are revealed, with the unprejudiced eye of experience, to investigate them scientifically in every point that is of importance to the physician, and to collect them into one whole, is the province of medical psychology."†

The human mind must be studied in connexion with the material conditions of the encephalon, since it is upon the vesicular matter of the encephalic ganglia that the mind is dependent for the manifestation of all its activities in this life. And it has long been my own settled conviction that the metaphysician can make little progress independently of the physiologist, and that it is to the medical philosopher and physiologist we are to look for the most valuable contributions to the science of mind. To be reminded of what they have done, we have only to recall the names of Locke, Hartley, Brown, &c.

The expressive language of Dugald Stewart, in reference to

* "Dr. Hartley's 'Observations on Man,'" says Dr. Southwood Smith, "is a work which does honour to human nature. One feels proud to belong to the same order of intelligences with the mind which could compose it. All that relates to the Law of Association, and the whole of the 2nd volume, can never be perused without making the reader better acquainted with himself and with his duties, and more in love with his fellow-beings and with his Creator. The conclusion, on the Final Happiness of all Mankind, is truly worthy of the philosopher, the philanthropist, and the Christian."—*Illustrations of the Divine Government*, by Dr. Southwood Smith. London. 1822. Pp. 445.

† "Medical Psychology." Translated and published by the Sydenham Society. 1847.

Locke, in his admirable dissertation on the progress of philosophy, admits of general application :—

"No science," says he, "could have been chosen more happily calculated than *medicine* to prepare such a mind as that of Locke for the prosecution of those speculations which have immortalized his name; the complicated and fugitive, and often equivocal phenomena of disease, requiring in the observer a far greater portion of discriminating sagacity than those of physic strictly so called."

The praise which our English Hippocrates, Sydenham, the greatest authority of his time, bestows on the medical skill of Locke, affords a brilliant proof of the high estimation which his acquirements in the science of medicine, his penetrating judgment, as well as his many private virtues, had procured for him from all who knew him. In the dedication prefixed to Sydenham's "Observations on the History and Cure of Acute Diseases," published in 1676, he boasts of the approbation bestowed upon his method by Mr. John Locke, who, to borrow Sydenham's own words, "had examined it to the bottom; and who, if we consider his genius and penetrating and exact judgment, has scarce any superior, and few equals now living."

"Nostri præterea quam huic meæ methodo suffragantem habeam, qui eam intimeus per omnia perspexerat utrique nostrum conjunctissimum dominum Joannem Locke; quo quidem viro, sive ingenio judicioque acri et subacto, sive etiam antiquis, hoc est, optimus moribus, vix superiorem quenquam, inter eos qui nunc sunt homines repertam, iri confido, paucissimus certe pares."*

In conclusion, I may reiterate what I have elsewhere said:—

"To Locke we are indebted for dispelling the mysticism of the schoolmen. Freed from the tyranny of ancient names, and regardless alike of the Stagyrite and his categories, he discarded the syllogism, and instituted a searching analysis of the phenomena of thought. In the metaphysical world, like the immortal Newton in the mathematical world, he stands forth pre-eminent. No age or nation ever produced two greater luminaries of science. They live in the veneration of their countrymen, and are borne down the stream of time with a reputation ever gathering, and with the triumphs of a distinction that will never die."†

In this essay I have alluded to the illustrious Gall, and his able associate, Spurzheim, as being the founders of physiological phrenology; but it is to Unser and Procraska that the honour is due for having accurately defined the boundaries of the sensorium commune. And since their time, and both in this country and abroad, there have been many labourers in the field, and much

* *Vide* Lord King's "Life of Locke."

† "Physiological Psychology." Commentary, p. 17, *ante cit.*

has been effected towards a better understanding and a more exact knowledge of the functions and special endowments of the nervous centre of the encephalon.

We must not forget the labours and researches of Rolando and Bellengeri, and still more recently of Matteucci, in Italy,—of Magendie, Serres, Des Moulins, and Flourens in France,—of Tiedemann in Germany,—and of Retzius in Sweden, &c. And while the discovery of Sir Charles Bell marks a new era in physiological science, the researches of his contemporaries, Shaw, Mayo, &c., and in our own day those of Swan, Owen, Marshall Hall, Solly, Todd, Carpenter, &c., have thrown a flood of light upon the subject. Among living physiologists, Dr. Carpenter has done more than any other man to specialize the functions of the nervous centres of the encephalon, and through comparative anatomy, by analytical reasoning and strict induction, to advance our knowledge of the physiological psychology of man.

With the labours and researches of Gall and Spurzheim the name of Mr. George Combe is indelibly associated, and will be held in enduring remembrance. His last work, "On the Relation between Science and Religion,"* is worthy of the author of the "Constitution of Man;" for, to use the words of one to whom I am under great obligations, "Every system of philosophy rests in God, as its highest idea and its final aim. To see the DIVINITY as the beginning, the middle, and the end of all things, is the culminating point of *all human thought.* Thus it is the goal, not only of providence, not only of redemption, but also of the no less divine laws of reason itself, that GOD *should be all in all.*"†

---

### ART. VI.—MIND AND BODY.

* "The Relation between Science and Religion," by George Combe. Edinburgh. 1857. Fourth Edition.

† Morel's "Psychology," p. 253, *ante cit.* One of the most valuable contributions to the Science of Mind which we have in our language.

